# Double Bolus Thrombolysis for Suspected Massive Pulmonary Embolism during Cardiac Arrest

**DOI:** 10.1155/2015/367295

**Published:** 2015-11-17

**Authors:** Gerard O'Connor, Gareth Fitzpatrick, Ayman El-Gammal, Peadar Gilligan

**Affiliations:** ^1^Department of Emergency Medicine, Mater Misericordiae University Hospital, Eccles Street, Dublin 7, Ireland; ^2^Department of Emergency Medicine, Beaumont Hospital, Beaumont Road, Dublin 9, Ireland

## Abstract

More than 70% of cardiac arrest cases are caused by acute myocardial infarction (AMI) or pulmonary embolism (PE). Although thrombolytic therapy is a recognised therapy for both AMI and PE, its indiscriminate use is not routinely recommended during cardiopulmonary resuscitation (CPR). We present a case describing the successful use of double dose thrombolysis during cardiac arrest caused by pulmonary embolism. Notwithstanding the relative lack of high-level evidence, this case suggests a scenario in which recombinant tissue Plasminogen Activator (rtPA) may be beneficial in cardiac arrest. In addition to the strong clinical suspicion of pulmonary embolism as the causative agent of the patient's cardiac arrest, the extremely low end-tidal CO_2_ suggested a massive PE. The absence of dilatation of the right heart on subxiphoid ultrasound argued against the diagnosis of PE, but not conclusively so. In the context of the circulatory collapse induced by cardiac arrest, this aspect was relegated in terms of importance. The second dose of rtPA utilised in this case resulted in return of spontaneous circulation (ROSC) and did not result in haemorrhage or an adverse effect.

## 1. Introduction

Acute pulmonary embolism is a common disease with well-recognised morbidity and mortality [1–3]. It can present with variable, often nonspecific signs and symptoms, and this often leads to delayed diagnosis [4–6]. The TROICA study argued against the indiscriminate use of lysis in those in cardiac arrest [7]. Emergent thrombolysis is however being increasingly utilised for those with immediately life-threatening complications of pulmonary embolism [8–10].

In this regard, contemporary guidelines [11] suggest administration of thrombolysis for high-risk patients with pulmonary embolism (shock and/or hypotension present) and intermediate risk patients with pulmonary embolism where haemodynamic decompensation is present (as a result of evidence of both RV dysfunction—by echocardiography or CT angiography—and elevated cardiac biomarker levels in the circulation).

High level evidence in respect of thrombolysis of PE during cardiac arrest is lacking. While there are case reports and case series that describe successful resuscitation following administration of systemic thrombolytic therapy during cardiac arrest (from suspected PE) [12–16], there is also literature arguing against its use [17, 18]. This is in addition to the arguments that arise as a result of the publication bias of successful case reports and case series. Guidelines—including Class I recommendation from the European Society of Cardiology [11]—advocate proceeding with lysis in cardiac arrest associated with confirmed PE and also proceeding with lysis in cases of cardiac arrest associated with suspected PE [19, 20].

The most frequently used emergency thrombolysis dosing regimen for PE in cardiac arrest remains the prototypical 2003 British Thoracic Society regime of alteplase 50 mg intravenous (IV) bolus [21]. It is unclear on the optimum approach in those in which first-dose bolus systemic thrombolysis fails to achieve return of spontaneous circulation (ROSC). Options may involve a decision to administer a second bolus of thrombolysis, catheter directed thrombolysis, or other intervention. Extra-corporeal life support [22, 23] and surgical embolectomy [11, 20, 24–26] are treatment options in massive PE, although the decision on these options becomes even more complicated in those in cardiac arrest. This case of successful resuscitation following double dose thrombolysis should help to inform the decision making process for those facing a similar dilemma in the future.

## 2. Case

A 39 year-old gentleman presented to the Emergency Department with a two-day history of pleuritic chest pain, lethargy and associated symptoms of progressively increasing shortness of breath (now occurring with minimal exertion). This occurred on a background of more long-standing non-specific lethargy. There was a history of a recent long-haul flight from Nigeria to Ireland one week previously. At presentation, he exhibited a tachycardia of 116 beats per minute, blood pressure of 131/94 mmHg, and a respiratory rate of 22 breaths per minute.

An electrocardiogram revealed a sinus tachycardia with symmetrical T wave inversion in praecordial lead V3. Arterial blood gas analysis showed a PaO_2_ of 7.5 kPa, PaCO_2_ of 3.8 kPa, pH 7.47, and an oxygen saturation of 89%. A D-dimer assay performed at triage was significantly elevated at 10.5 mg/L. Given the working diagnosis of probable pulmonary embolus (high-risk pretest probability), therapeutic low-molecular-weight heparin (Enoxaparin 120 mg subcutaneously) was administered prior to emergent Computed Tomographic Pulmonary Angiography (CTPA).

Two hours later, while awaiting emergent CTPA, the patient collapsed and was found to be in cardiac arrest. Cardiopulmonary resuscitation was promptly initiated for pulseless electrical activity (PEA). Intubation with a cuffed oroendotracheal tube (COETT) was achieved without interruption of chest compressions. Despite primary confirmation of COETT placement, end-tidal CO_2_ was not detected initially. Subxiphoid ultrasound—performed during brief interruption of chest compressions—did not reveal a dilated right side of heart. Despite this, given the overall clinical picture at this juncture, a presumptive diagnosis of massive or saddle pulmonary embolus was made.

Along with conventional ACLS adrenaline therapy, rtPA (alteplase) 50 mg was promptly administered. Despite continuing high quality chest compressions and a gradual rise in quantitative end-tidal CO_2_, no cardiac output was detected after twenty minutes. A decision was taken to administer a second bolus of rtPA (alteplase) 50 mg. Ten minutes subsequent to this and following on-going advanced life support, return of spontaneous circulation (ROSC) was achieved with an initial non-invasive blood pressure of 144/50 mmHg.

Standard post-ROSC resuscitation care was instituted and this gentleman was admitted to the intensive care unit. A CTPA demonstrated multiple bilateral pulmonary emboli. He was continued on Enoxaparin and bridged to Warfarin once critical care stability was achieved. No major (or minor) bleeding was observed during this gentleman's hospital stay. The patient ultimately recovered to hospital discharge with a Glasgow Outcome Score of 4, secondary to watershed cerebellar infarcts.

## 3. Discussion

A number of trials and guidelines address the issue of thrombolysis during a massive pulmonary embolism and sub-massive pulmonary embolism. There are few specific guidelines which directly address the issue of thrombolysis during cardiac arrest in those with (suspected) massive pulmonary embolism [27], that is, fulminant cases.

The British Thoracic Society (BTS) recommends a bolus of 50 mg alteplase for massive PE [21] and states that this may be* “instituted on clinical grounds alone if cardiac arrest is imminent.”* The American Heart Association seems to recommend a two-hour infusion of 100 mg of alteplase in those with haemodynamic compromise (though they do not explicitly address the issue of cardiac arrest) [28]. The 2014 European Society of Cardiology Guidelines [11] recommend a dose of 100 mg rtPA over 2 hours or 0.6 mg/kg over 15 minutes [29], though again they are not explicit regarding the approach in cardiac arrest. We felt that a prolonged infusion might not represent the best approach in the situation presented, given the understandable exigencies of cardiac arrest.

There are numerous case reports and case series describing survival post thrombolysis in cardiac arrest caused by PE. Er et al. [30] retrospectively studied 104 patients in whom thrombolysis was administered for presumptive PE cardiac arrest. ROSC was achieved in 40 patients with survival to hospital discharge in 19 patients. Both ROSC and survival to hospital discharge were associated with earlier initiation of thrombolysis. Patients in this trial were treated with bolus dose rtPA with an average dose of 80.5 ± 24 mg. Janata et al. [31] describe a retrospective review of cardiac arrest patients with the cause of arrest secondary to massive PE. Sixty-six patients were reviewed with 36 of these patients treated with rtPA. They administered rtPA as a bolus of 0.6–1.0 mg rtPA/kg body weight up to a maximum of 100 mg of rtPA. Return of spontaneous circulation showed a trend towards improvement in the rtPA group (67% versus 43%, *P* = 0.06) as well as survival to discharge (19% versus 7%, *P* = 0.15).

Once thrombolysis is initiated for a suspected PE in cardiac arrest, guidelines suggest that CPR should be continued for at least 60–90 minutes [13, 32–36].

Domino or double dose thrombolysis also appears in the literature. In the sentinel study by Böttiger et al. [37], 90 patients were assigned to intervention (thrombolysis) or control arms of treatment for cardiac arrest. Those in the intervention arm received a bolus of 5000 IU of heparin with 50 mg rtPA after 15 min of unsuccessful CPR, with a repeat bolus of heparin and rtPA 30 minutes later if ROSC was not achieved. While there is no breakdown on the numbers receiving double dose thrombolysis, in this early study on thrombolysis in cardiac arrest they noted statistically significant increases in ROSC and survival to hospital admission in the thrombolysis group. Kürkciyan et al. [38] describe intervening with regimens of 100 mg rtPA (either as a 50 mg double bolus or as a bolus dose of 15 mg, followed by continuous infusion of 85 mg over 90 minutes). In this intervention arm the two survivors to hospital discharge received double bolus doses. Similarly Fengler and Brady [39], in their suggested treatment algorithm, advocate a repeat bolus of alteplase 50 mg if ROSC is not achieved after 15 minutes after the first dose. In our case, we cannot discount that, despite achievement of ROSC after the second bolus, this success might be better explained by the haemodynamic improvements brought about by the first bolus [40].

There is general consensus that thrombolysis should be considered in cardiac arrest where pulmonary embolism is strongly suspected. The exact dosage and timing of fibrinolysis remain to be clarified [7, 19, 37, 41], though there does appear to be a trend towards improved survival in those in whom intervention is initiated at an earlier juncture. This is seen in the study by Er et al. [30] in which those patients who survived to hospital discharge benefitted from earlier initiation of lysis compared to all other patients (11.0 ± 1.3 versus 22.5 ± 0.9 min; *P* < 0.001). The issue of risks and benefits in terms of haemorrhage remains a major consideration and while the principle of* primum non nocere* is more difficult to weigh in those in cardiac arrest, it should be remembered that the bleeding risks remain significant. Evidence would suggest that thrombolysis does not seem to be unduly associated with catastrophic haemorrhage in this critically ill patient group [31, 42, 43]; nevertheless there are still recognised major haemorrhage rates and intracranial haemorrhage rates of up to 10.4% [44] and 3.6% [45] in contemporary prospective trials.

Low end-tidal CO_2_ (ETCO_2_) is seen both in cardiac arrest [46–48] and in massive pulmonary embolism [49–55]. Increases in ETCO_2_ are seen in recovery from both entities and have prognostic value in those in cardiac arrest in predicting the likelihood of ROSC [56]. The unrecordable levels seen at the outset of this case were assumed to result from the absolute no flow through the pulmonary circulation rather than the low flow that is seen in cardiac arrest.

Common echocardiographic findings in massive pulmonary embolism include that of an enlarged right ventricle [57] which may be associated with a flattened interventricular septum (D-sign of interventricular septal shift) and the “McConnell” sign [58, 59]. These tests were originally described in those with massive PE (and were originally performed with transthoracic approaches rather than with subxiphoid views) so their validity and applicability in terms of positive and negative predictive value for patients in cardiac arrest are unknown. Therefore, the absence of the echocardiographic features should not be used to rule out PE as a cause of cardiac arrest [60].

In conclusion, this particular case describes a clinical scenario in which double dose thrombolysis was successfully used. A similar strategy might be contemplated in the future by emergency physicians dealing with cardiac arrest caused by massive pulmonary embolism.
